# The Phytochemical α-Mangostin Inhibits Cervical Cancer Cell Proliferation and Tumor Growth by Downregulating E6/E7-HPV Oncogenes and *KCNH1* Gene Expression

**DOI:** 10.3390/ijms24033055

**Published:** 2023-02-03

**Authors:** Lorenza Díaz, Samantha V. Bernadez-Vallejo, Rafael Vargas-Castro, Euclides Avila, Karla A. Gómez-Ceja, Rocío García-Becerra, Mariana Segovia-Mendoza, Heriberto Prado-Garcia, Galia Lara-Sotelo, Javier Camacho, Fernando Larrea, Janice García-Quiroz

**Affiliations:** 1 Departamento de Biología de la Reproducción Dr. Carlos Gual Castro, Instituto Nacional de Ciencias Médicas y Nutrición Salvador Zubirán, Mexico City 14080, Mexico; 2 Departamento de Biología Molecular y Biotecnología, Instituto de Investigaciones Biomédicas, Universidad Nacional Autónoma de México, Mexico City 04510, Mexico; 3 Departamento de Farmacología, Facultad de Medicina, Universidad Nacional Autónoma de México, Mexico City 04510, Mexico; 4 Laboratorio de Onco-Inmunobiología, Departamento de Enfermedades Crónico-Degenerativas, Instituto Nacional de Enfermedades Respiratorias Ismael Cosío Villegas, Mexico City 14080, Mexico; 5 Departamento de Farmacología, Centro de Investigación y de Estudios Avanzados del I.P.N., Mexico City 07360, Mexico

**Keywords:** α-mangostin, cervical cancer, KCNH1, HPV, E6, E7

## Abstract

Cervical cancer is the fourth most common cancer among women worldwide. The main factor associated with the onset and progression of this neoplasia is the human papillomavirus (HPV) infection. The HPV-oncogenes E6 and E7 are critical drivers of cellular transformation, promoting the expression of oncogenes such as *KCNH1*. The phytochemical α-mangostin (AM) is a potent antineoplastic and antiviral compound. However, its effects on HPV oncogenes and *KCNH1* gene expression remain unknown. This study evaluated the effects of AM on cell proliferation, cell cycle distribution and gene expression, including its effects on tumor growth in xenografted mice. AM inhibited cell proliferation in a concentration-dependent manner, being the most sensitive cell lines those with the highest number of HPV16 copies. In addition, AM promoted G1-cell cycle arrest in CaSki cells, while led to cell death in SiHa and HeLa cells. Of interest was the finding of an AM-dependent decreased gene expression of E6, E7 and *KCNH1* both in vitro and in vivo, as well as the modulation of cytokine expression, Ki-67, and tumor growth inhibition. On these bases, we suggest that AM represents a good option as an adjuvant for the treatment and prevention of cervical cancer.

## 1. Introduction

Cervical cancer is still one of the primary diagnosed neoplasia in women worldwide [1]. The primary risk factor for developing cervical cancer is the persistent infection with high-risk human papillomavirus (HPV) subtypes, which are detected in about 99% of cervical malignant lesions, HPV16 and HPV18 subtypes being responsible for at least 60% and 15% of all of them, respectively [2,3,4]. Onset and progression to cervical cancer entirely depend on the two major HPV oncogenes E6 and E7, which cooperate to keep HPV DNA replication, apoptosis evasion, genome instability and cell immortalization [2,5]. E6 abrogates P53 function, while E7 targets pRB for proteasomal degradation [2]. Interestingly, an additional carcinogenic mechanism of E6 and E7 includes the upregulation of the oncogenic potassium voltage-gated channel subfamily H member 1 (*KCNH1*) gene, that encodes the ether-à-go-go 1 (EAG1, Kv10.1) potassium channel [6]. On the contrary, the transfection of pRb into cervical cancer cells downregulates this channel [7]. *KCNH1* is widely expressed in the central nervous system and is practically undetected in peripheral tissues. However, this channel is upregulated in malignant and premalignant lesions and is associated with tumor progression [8,9]. Consequently, the inhibition of *KCNH1* expression and/or activity results in decreased in vitro and in vivo cancer cell proliferation [10,11]. In cervical cancer, *KCNH1* is considered a potential tumor marker and therapeutic target [12,13].

Despite the successful introduction of prophylactic vaccines against high-risk HPV subtypes, cervical cancer is still a significant public health problem and one of the most common causes of cancer death in women worldwide [1,2]. Thus, new therapeutic strategies are needed, and research into new anticancer drugs from natural sources is gaining great interest [14]. Among the natural compounds studied, the xanthone α-mangostin (AM), a phytochemical obtained from the pericarp of mangosteen fruit, exhibits broad biological activities, with outstanding antineoplastic [15] and anti-viral effects [16,17,18,19,20,21,22,23,24]. The antitumor effects of AM, have been widely evaluated in different malignancies [15], including cervical cancer [25,26,27,28]. Additionally, the antiviral activity of AM, has been described against rotavirus [17], hepatitis C virus [24], dengue virus [18,19,20,21], chikungunya virus [23], and SARS-CoV-2 [22]. However, the AM effects on *KCNH1* expression and HPV *E6/E7* regulation remain unknown. Therefore, because of the causative and relevant role of HPV in cervical cancer and the need for novel therapeutic approaches, herein we aimed to evaluate the antineoplastic and antiviral effects of AM in both in vitro and in vivo models of cervical cancer, focusing on the potential mechanism underlying these effects.

## 2. Results

### 2.1. AM Inhibits Cervical Cancer Cell Proliferation

**Figure 1:**
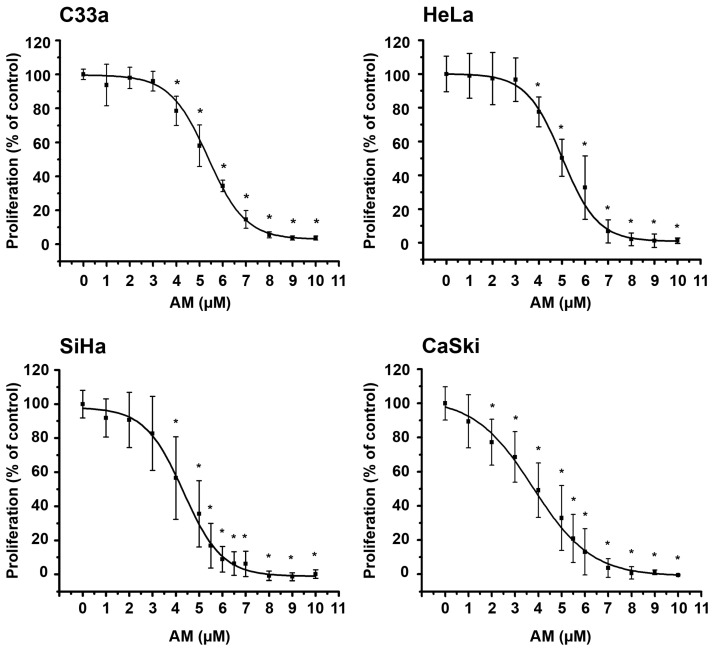
Effect of AM on cervical cancer cell proliferation. The antiproliferative effects of α-mangostin (AM) were tested in a panel of cervical cancer cells with different HPV viral load. The antiproliferative effect of AM was evaluated using a range of concentration from 1 to 10 µM. The phytochemical significantly inhibited cancer cell proliferation in a concentration-dependent manner. Results are depicted as the mean ± SD of at least three independent experiments. The data from the vehicle-treated cells were normalized to 100%. * *p* < 0.05 vs. vehicle.

As an in vitro model of cervical cancer, we used a panel of three cell lines with different HPV subtypes, including: HeLa (10 to 50 copies of HPV18 per cell), SiHa (1 to 2 copies of HPV16 per cell), and CaSki (about 600 copies of HPV16 per cell), as well as HPV-negative C33a cells. To study the effect of AM on the proliferation of these cells, incubations in the presence or the absence of this compound were carried out over 6 days, and then, cell proliferation was studied by the sulforhodamine B (SRB) assay [29]. The results showed that AM inhibited cervical cancer cell proliferation in a concentration-dependent manner in all tested cell lines (Figure 1).

Based on the concentration-response curves, the inhibitory concentration values at 50% (IC_50_) of AM in each cell line were calculated (Table 1). Consistently with the IC_50_ values, the phytochemical’s most significant growth inhibitory effect was observed in CaSki cells, followed by SiHa, HeLa, and finally, C33a cells. Interestingly, the most sensitive cells had the highest number of HPV copies.

### 2.2. AM Differentially Modified the Cell Cycle Distribution of Cervical Cancer Cells Depending on the HPV Status

**Figure 2:**
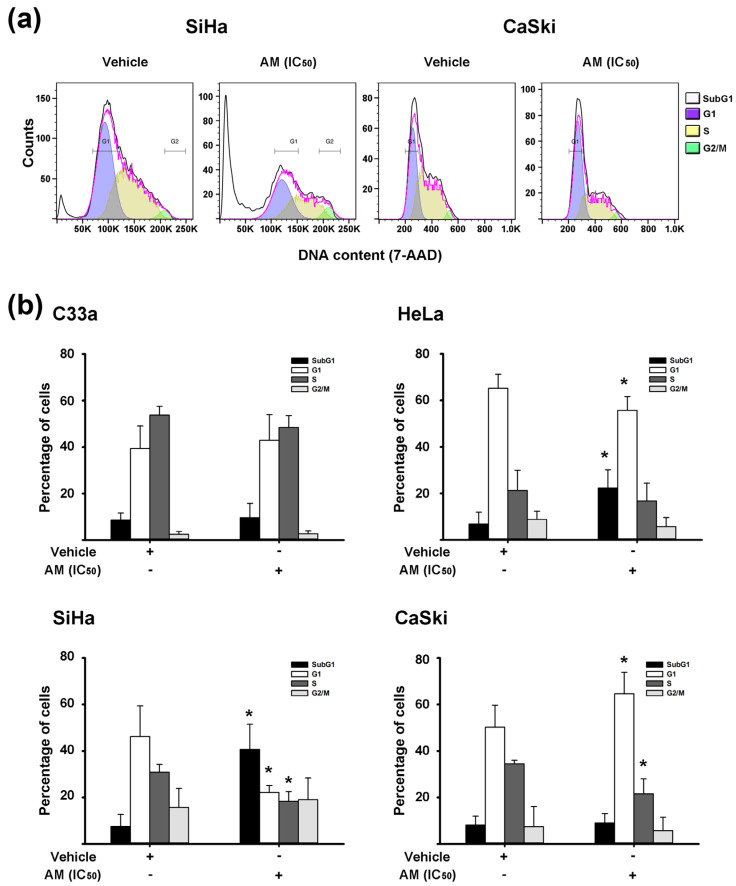
Effects of AM on the cell cycle distribution of cervical cancer cells. Cells were treated with α-mangostin (AM) at its respective inhibitory concentration at 50% (IC_50_), and after 48 h, the cell cycle distribution was analyzed by flow cytometry. (**a**) Representative flow cytometry histograms of SiHa and CaSki cells treated with either vehicle or AM. The G1-cell cycle phase is shown in purple, the S-region in yellow, and the G2/M in green. The SubG1 subpopulation, indicating cell death, is represented by the white color. The pink line shows how well the mathematical model Dean-Jett-Fox fits the histogram (black line). (**b**) Graphics show the mean percentage of cells ± SD per cell cycle-phase in three replicates of at least three independent experiments. * *p* < 0.05 vs. vehicle group.

To discern whether the AM antineoplastic effects were cytostatic or cytotoxic, we analyzed the cell cycle distribution of cervical cancer cells treated with the phytochemical for 48 h at their respective IC_50_ values. AM increased the frequency of cells in the SubG1 cell phase, while decreased the proportion of cells in the G1 phase in HeLa and SiHa cell lines. Additionally, in SiHa cells there was a decreased number of S-phase cells. Regarding CaSki cells, AM promoted cell cycle arrest in the G1-phase, accompanied by a decreased number of S-phase cells. In C33a cells, the xanthone did not exert any change in the cell cycle profile (Figure 2).

### 2.3. AM Down-Regulated E6/E7-HPV16 Oncogenes and KCNH1 Gene Expression

Because the most sensitive cells to the antiproliferative effects of AM were SiHa and CaSki, which express variable copies of HPV16 sequences, the effect of the natural compound on *E6*- and *E7*-HPV16 gene expression was evaluated. An inhibition of *E6*- and *E7*-HPV16 oncogenes expression was achieved at 10 µM of AM in both cell lines. However, a stronger and statistically significant inhibition was observed in CaSki cells (Table 2).

Based on the role of *KCNH1* in cancer cell proliferation and considering that the transformation of normal keratinocytes with HPV *E6* and *E7* oncogenes results in increased *KCNH1* gene expression [6], we evaluated the effect of AM upon this gene. Notably, AM significantly inhibited *KCNH1* gene expression in a concentration-dependent manner in CaSki cells, while in SiHa cells a non-significant inhibitory trend was observed (Table 3).

### 2.4. AM Modulated Cytokines and Vimentin Gene Expression

**Figure 3:**
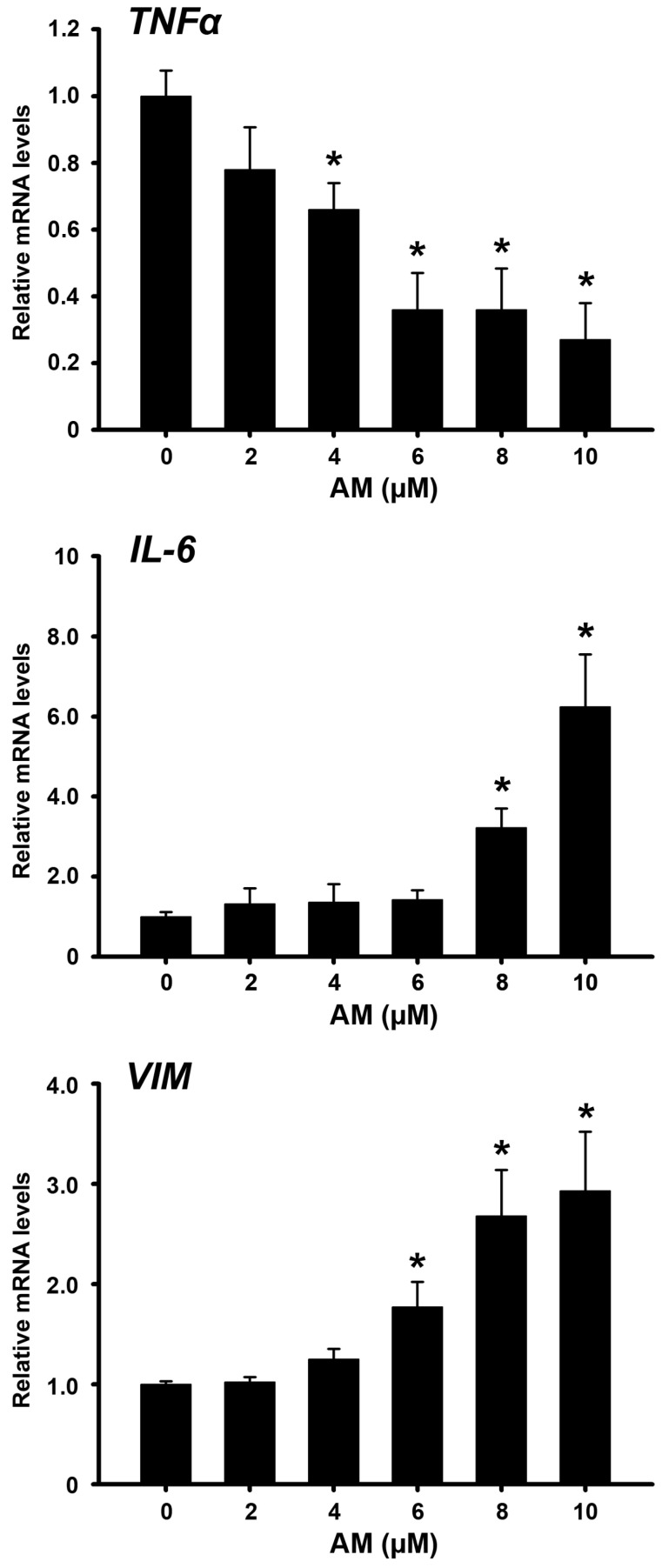
AM modulates cytokines and vimentin gene expression in CaSki cells. α-mangostin (AM) significantly differentially regulated tumor necrosis factor α (*TNFα*), interleukin-6 (*IL-6*) and vimentin (*VIM*) gene expression. The results are the mean ± SEM of relative *TNFα, IL-6,* and *VIM* mRNA levels after normalizing against the housekeeping gene glyceraldehyde 3-phosphate dehydrogenase (*GAPDH*) mRNA expression. N ≥ 3 independent experiments. * *p* < 0.05 vs. vehicle-treated cells.

Interestingly, it is known that AM reduces rotavirus infection by improving the innate immune response against viral infection [17]. Additionally, inflammation-induced carcinogenesis, such as persistent HPV cervical infection, includes the participation of cytokines, such as tumor necrosis factor-alpha (TNFα), interleukin-6 (IL-6), and interleukin-1 beta (IL-1β). Therefore, we evaluated the effect of AM on the gene expression of these cytokines at the transcriptional level. Once again, we observed consistent changes in CaSki cells only. AM significantly inhibited *TNFα* gene expression while increasing that of *IL-6* in a concentration-dependent manner (Figure 3). Regarding *IL-1β* gene expression, a significant increase was only observed with AM at 8 µM, which doubled the cytokine expression compared with vehicle (data not shown).

On the other hand, it has been reported that vimentin prevents the internalization of HPV16, and its expression is inversely correlated with the viral infection [30]. Therefore, we evaluated the effect of AM on vimentin gene expression. The phytochemical increased vimentin gene expression in a concentration-dependent manner (Figure 3).

### 2.5. Antitumoral Effects of AM in an In Vivo Cervical Cancer Model

**Figure 4:**
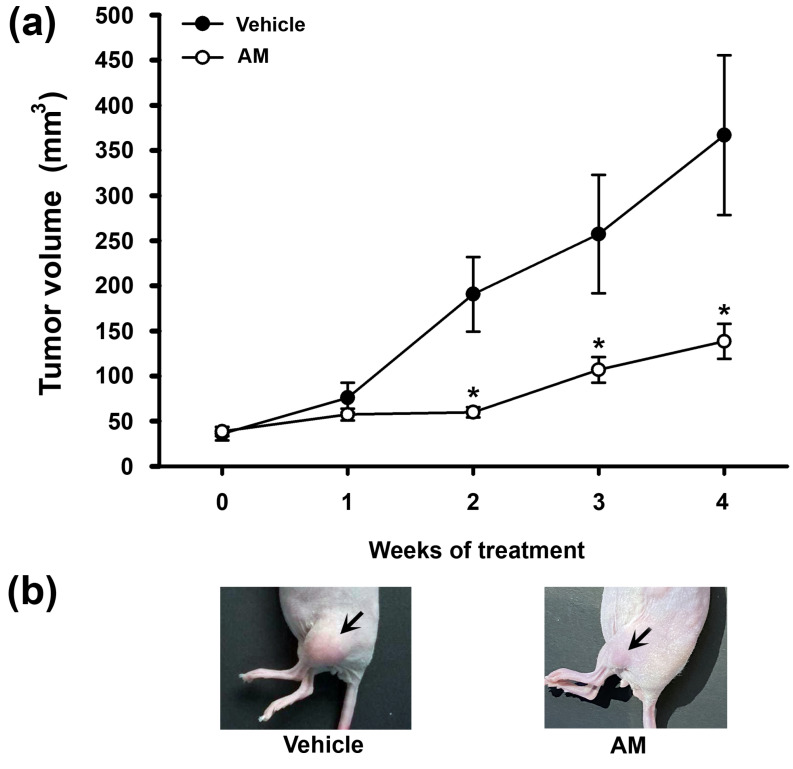
AM administration slows cervical tumor growth. SiHa cervical cancer cells were subcutaneously xenografted (2 × 10^6^ cells) into the lower limb of each mouse. Mice were randomly divided into two groups when the tumors reached a palpable mass of ~5 mm in diameter, and received either vehicle (0.1% of DMSO/day, black circles) or α-mangostin (AM, 8 mg/kg of body weight/day, white circles) in the drinking water for four weeks. (**a**) The tumors were measured every week with a caliper, and the tumor volume was estimated. The results are the mean ± SEM of tumor volume. (**b**) Representative pictures depict the tumor’s final size at the end of the treatment. In the vehicle and AM-treated groups, 9 and 10 mice were included, respectively. * *p* < 0.05 vs. vehicle.

The observed in vitro effects of AM on cervical cancer cell proliferation prompted us to further explore its in vivo effect on the tumor growth of xenografted cervical cancer cells. First, we evaluated the tumorigenicity of CaSki and SiHa cells. Under this tumor model, CaSki cells were not tumorigenic (*n* = 11 mice). On the contrary, as shown in Figure 4, SiHa cells readily formed tumors that constantly increased in size throughout 4 weeks in the vehicle-treated group of mice. The administration of AM significantly reduced tumor growth starting from the second week of treatment compared with vehicle-treated mice. It is notable that the final body weights were not significantly different between the vehicle and AM treated mice (22.67 ± 2.11 g and 22.56 ± 2.89 g, respectively).

**Figure 5:**
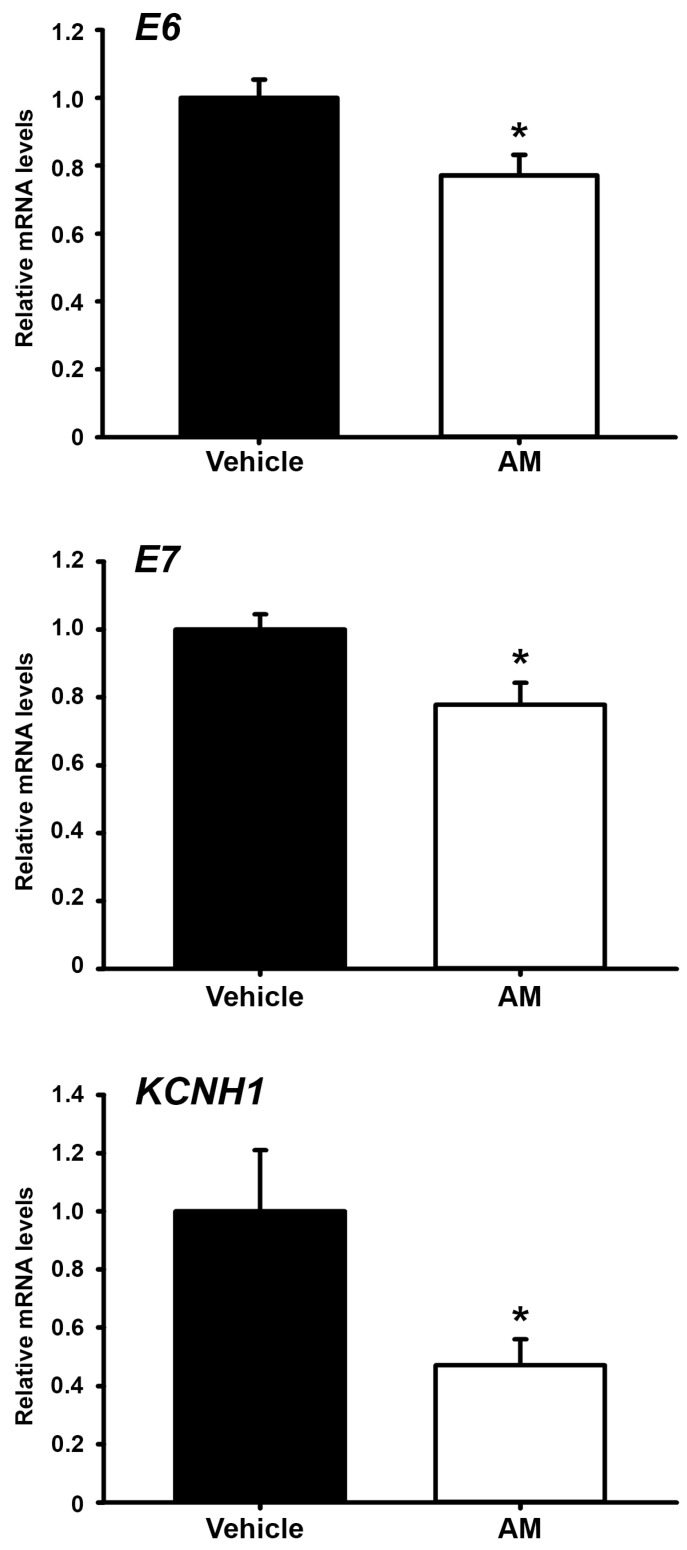
AM decreases *E6/E7*-HPV16 oncogenes and *KCNH1* tumor gene expression. After four weeks of treatment with vehicle (0.1% DMSO, black bars) or α-mangostin (AM, 8 mg/kg of body weight/day, white bars), the mice were euthanized and the tumor tissue was collected and submitted to RT-qPCR experiments. Gene expression level was normalized against the housekeeping gene RPL32. The results are the mean ± SEM of at least nine tumors per treatment group. * *p* < 0.05 vs. vehicle.

Based on the in vitro effects of AM on the *E6/E7* HPV oncogenes and *KCNH1* gene expression, we evaluated these effects on the tumor tissue from xenografted SiHa cervical cancer cells. The analysis demonstrated that AM significantly inhibited the gene expression of the *E6* and *E7* HPV oncogenes and *KCNH1* in the tumor mass (Figure 5).

**Figure 6:**
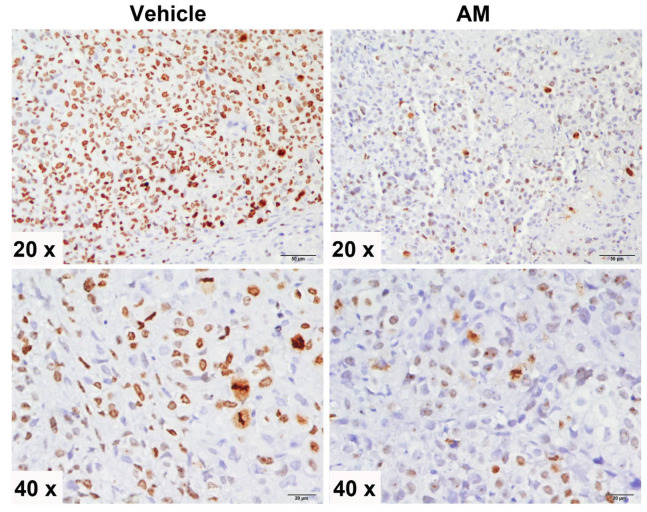
Immunohistochemical analysis of Ki-67 in the tumor tissue of vehicle and AM-treated mice. SiHa cervical cancer cells were subcutaneously xenografted into the lower limb of each mouse. Mice were randomly divided into two groups: vehicle-treated and α-mangostin (AM) -treated. After four weeks of treatment, the mice were euthanized and the tumor tissue was collected and fixed in formaldehyde for immunohistochemical staining. The proliferation marker Ki-67 was expressed in a lesser degree and by fewer tumoral cells of mice treated with AM for 4 weeks, as compared to controls. Representative 20× and 40× magnification photographs are shown for each group.

As an additional clinically relevant parameter, we studied the effect of AM upon the proliferation marker Ki-67 in the tumor mass. The vehicle-treated mice had tumors with less Ki-67 positivity, compared with AM-treated mice (Figure 6).

## 3. Discussion

Despite effective prophylactic vaccines against the high-risks HPVs and screening tests, cervical cancer remains the fourth most common cancer in women worldwide with over 600,000 new cases annually with half resulting in death [1], which occurs mainly in developing countries. Therefore, it is necessary to strengthen the ongoing research and improve the current treatments for cervical cancer patients. On this matter, preventive and therapeutic strategies involving phytochemicals are considered very useful due to their low cost and minimal undesirable effects [31]. Regarding this, the natural compound AM exhibits various pharmacological properties, including antioxidant, cardioprotective, antiallergic, antifungal, antibacterial, antiinflammatory, antiviral, and antineoplastic effects [32]. In this study, we aimed to evaluate the potential mechanisms underlying the last two properties of AM in cervical cancer, since HPV chronic infection is the leading risk factor for the development of this pathology.

We found that AM, inhibited in a concentration-dependent manner, cervical cancer cell proliferation, whose responsiveness was as follows: CaSki > SiHa > HeLa > C33a. This agrees with previous studies, where the antiproliferative effect of AM was observed in HeLa and SiHa cells [25,26,28]. Notably, in our research, the most AM sensitive cells were those with the highest number of HPV copies of the most carcinogenic HPV16 subtype, specifically, SiHa and CaSki cells. Therefore, we decided to evaluate the effect of AM on *E6*-HPV16 and *E7*-HPV16 gene expression in these cells. We focused on these oncogenes considering that their expression is tightly related to oncogenic transformation of epithelial cells. Moreover, E6 and E7 work together to control the cell cycle, inducing cell proliferation and immortalization; thus, representing attractive therapeutic targets. In this regard, herein we show for the first time that AM inhibited *E6*-HPV16 and *E7*-HPV16 gene expression under the range of concentrations at which the phytochemical inhibited dengue virus production and cytokine/chemokine expression [18,19,20,21]. These effects partially explain the reduced cell proliferation after treating the cervical cell lines with AM. Similarly, AM interferes with different viral infectious mechanisms, including the chikungunya virus infection and replication, possibly by interacting with multiple target proteins of the virus [23]. Likewise, in in silico models, it has been demonstrated that AM interacts with the main protease enzyme of SARS-CoV2, which might result in enzyme inhibition [22]. Furthermore, AM has been shown to suppress hepatitis C virus genome replication, down-regulating viral proteins expression [24]. Interestingly, AM also reduces the rotavirus infectivity, improving the innate immune response against the viral infection [17]. As described, part of the phytochemical’s antiviral mechanism involves the improvement of the immune response through cytokine modulation. Interestingly, treating dengue virus infected HepG2 cells with AM decreased *TNFα* and *IL-6* gene expression at concentrations higher than 10 µM [18]. Particularly, TNFα is involved in the maintenance and homeostasis of the immune system, inflammation, and host defense. However, this cytokine is also involved in pathological processes such as chronic inflammation, auto-immunity, and neoplastic diseases. Consequently, TNFα may be a good therapeutic target against cervical cancer [33]. In this study, we found that in the CaSki cells, AM significantly inhibited *TNFα* gene expression in a concentration-dependent manner, suggesting that this anti-inflammatory effect is part of the mechanisms involved in its anti-cancer activity.

Contrary to *TNFα*, *IL-6* was significantly upregulated by AM in our cultured CaSki cells. In cervical cancer patients, high serum levels of IL-6 have been associated with adverse prognoses [34]. Considering that silencing of *E6*-HPV has been shown to reduce *IL-6* expression, and tumor formation in SiHa and HeLa cells [35], we expected that the observed AM-dependent down-regulation of *E6/E7* expression would be linked to reduced IL-6 expression, but this was not the case. Maybe, this cytokine is involved in improving the anti-viral response. Further studies are needed to elucidate the biological meaning of these results. Nevertheless, it has been reported that HPV-dependent IL-6 secretion by tumor cells increases radio sensitivity in some cancers, thereby improving treatment efficacy [36].

Vimentin is an intermediate filament protein exerting different functions related to the cytoskeleton structure and cellular processes such as cell division and migration. However, it has complex implications in pathophysiology, and plays an important role in the epithelial-mesenchymal transition in cancer cells, making epithelial cells lose their polarity and their adhesive properties, thus, increasing the invasiveness of tumor cells [37]. On the other hand, in certain malignancies such as endometrial cancer, the lack of vimentin expression has been correlated significantly with lymph node metastasis, deep myometrium invasion, lymph vascular space invasion, advanced stages (III and IV), and higher tumor grade. Indeed, vimentin-negative patients had poorer overall survival compared with vimentin-positive patients [38]. Additionally, it has also been described an interesting participation of vimentin against certain viral infections [37]. For instance, vimentin is known to obstruct the translocations of viral ribonucleoproteins to the nucleus [39]. Similarly, vimentin has shown to interact with dengue virus at the replication complex, interfering with RNA replication [37]. Furthermore, and of utmost importance for our research, vimentin has been reported to prevent the internalization of HPV16, while its expression has been inversely correlated with viral infection [30]. In fact, extracellular vimentin (surface and soluble) hampers the initial steps of HPV16 infection, identifying vimentin as an HPV restriction factor [30]. Given that virtually all cervical cancer cases are attributable to high-risk HPV types infection, our finding that AM significantly increased vimentin gene expression in cervical cancer cells suggests that this xanthone could help to prevent HPV viral infection.

Based on the above observations, AM could act as an excellent agent to prevent and treat cervical cancer since it reduces the *E6/E7* HPV gene expression, and induces a factor that restricts HPV internalization, namely vimentin, while diminishing TNFα gene expression and cervical tumor growth.

On the other hand, one of the most studied ion channels in cancer is the *KCNH1,* a voltage-gated potassium channel expressed in most human tumors [40]. In this study, we report for the first time that AM inhibited *KCNH1* gene expression both in vitro and in vivo, which adds to the described potential mechanism by which this xanthone inhibits cervical cancer cell proliferation. Similarly, AM has been shown to inhibit the activity of other ion channels expressed in immune cells, including ORAI1, Kv1.3, and KCa3.1 [41], some of which are also involved in cancer cell proliferation [42].

Regarding AM effects on the cell cycle distribution of HeLa and SiHa cells, we found that the phytochemical increased the cell number in the Sub G1-phase, which indicates cell death. Supporting our results, previous studies have shown that AM induces apoptotic cell death by increasing Bax and cytochrome c release, decreasing Bcl-2, and activating caspase-9/caspase-3 cascade as well as the ASKI/MKK3/6/p38 signaling pathway in HeLa and SiHa cervical cancer cells [26]. Moreover, we also found that in CaSki cells, AM promoted cell cycle arrest in the G1-phase, which is in accordance with *KCNH1* inhibition. Indeed, it has been shown that *KCNH1* inhibition promotes G1-phase cell cycle arrest [43]. Actually, E2F-binding to *KCNH1* could be a potential mechanism linking the effect of AM on HPV-oncogene regulation, cell cycle arrest, and *KCNH1* gene expression [44].

The AM in vitro effects herein studied encouraged us to explore its actions in a xenograft mice model. Tumor volume in xenografted SiHa cells was greater in the vehicle-treated group than in the AM-treated group, which was in accordance with Ki-67 positivity in tumor tissue.

Similar to our findings, other studies have also shown an antitumoral effect of AM [25,26,27]. Importantly, we did not observe changes in the mean body weights between the experimental and vehicle-treated groups, suggesting that the dose of AM used in this study was well tolerated during the period of time evaluated. In accordance with our observations, different clinical trials have evaluated the safety and tolerability profile of mangosteen products, showing in general no undesirable side effects [45,46,47,48].

Remarkably, the effect of AM upon *E6*-, *E7*-HPV16, and *KCNH1* expression in the tumor mass was similar to that observed in vitro in cultured cervical cancer cells. Accordingly, a prospective study on cancer and nutrition showed an inverse association between consuming fruits, vegetables, and nutrients with decreased risk of HPV infection, development of cervical intraepithelial neoplasia, and cervical cancer [49].

Cervical cancer development is highly associated with HPV infection. Herein we show the anti-HPV oncogene effects of AM, as well as additional potential mechanisms of its antineoplastic activity using different cervical cancer models. Therefore, we suggest that this xanthone represents an excellent candidate for a chemopreventive agent as well as an adjuvant for cervical cancer conventional treatment. Regarding the last, several preclinical studies have evaluated the antineoplastic effect of AM in conjunction with chemotherapeutic agents in different malignancies, including cervical cancer [25,27], supporting the concept that AM could act as an adjuvant agent for conventional chemotherapy.

## 4. Materials and Methods

### 4.1. Reagents

The following reagents were purchased from Sigma-Aldrich (St Louis, MO, USA): AM [chemically named: 1,3,6-trihydroxy-7-methoxy-2,8-bis (3-methyl-2-butenyl)-9h-Xanten-9-On], SRB, trichloroacetic acid (TCA), and 7-Aminoactinomycin D (7-AAD). DMSO was obtained from the American Type Culture Collection, ATCC (Manassas, VA, USA). All reagents for immunohistochemistry were obtained from Bio SB (Santa Barbara, CA, USA).

### 4.2. Cervical Cancer Cell Lines

Cervical cancer cell lines expressing *KCNH1* and/or HPV (Table 4) were used. The established human cervical cancer cell lines, C33a, HeLa, SiHa, and CaSki, were purchased from ATCC, and maintained following its indications. All experimental procedures were performed in supplemented DMEM-F12 medium conditioned with 100 units/mL penicillin plus 100 µg/mL streptomycin and 5% charcoal-stripped-heat-inactivated fetal bovine serum under standard cell culture conditions.

### 4.3. Proliferation Studies

One thousand cells per well were seeded in 96-well plates and after 24 h were incubated by sextuplicate in the presence of different AM concentrations (1–10 µM) or its vehicle (0.1% DMSO) for six days. Afterward, cell proliferation was measured using the SRB colorimetric assay [29]. Briefly, the cells were fixed with ice-cold TCA at 4 °C for one hour and air-dried, then the SRB (0.0057% in acetic acid) was added to each well and incubated at room temperature for one hour. The unbound dye was removed with three washes of acetic acid (1%), and then the dye attached to the proteins was extracted from the viable cells with an alkaline solution (10 mM Tris base, pH 10.5) and shaking. The absorbance was read at 492 nm in a microplate reader (Synergy HT Multi-Mode Microplate Reader, BioTek, Winooski, VT, USA). The IC_50_ values of AM were calculated analyzing the dose–response curves with the dose–response sigmoidal fitting function of the scientific graphing software Origin 9.0 (OriginLab Corporation, Northampton, MA, USA).

### 4.4. Cell Cycle Distribution

Cells were seeded in 6-well plates at 30,000–70,000 cells per well. After 24 h, the cells were incubated with the IC_50_ of AM for 48 h. After treatment, the cells were collected, washed with PBS, fixed in ethanol 70% *v*/*v*, and kept at −20°C. For cell cycle analyses, samples were washed twice with PBS pH 7.2 and incubated with Triton X-100 0.1% (*v*/*v*), and 7-AAD (1 µg/mL) in the dark at room temperature for 20 min. The DNA content was determined using a FACsCanto II flow cytometer (Becton Dickinson, CA, USA). At least 15,000 single events were acquired from the 7AAD-area vs. 7AAD-wide plot. For cell cycle analysis and subG1 peak detection, Flow-Jo software (Tree Star Inc., version 9.3.2) was used.

### 4.5. PCR Amplification

The effects of AM on mRNA expression of several genes were studied by extracting total RNA from treated cells using Trizol reagent (Life Technologies, Carlsbad, CA, USA). The concentration of RNA was estimated spectrophotometrically at 260/280 nm and 2 µg of RNA were reverse transcribed using the Maxima First Strand cDNA Synthesis kit (Thermo Fisher Scientific, Waltham, MA, USA). Gene expression of the housekeeping gene glyceraldehyde 3-phosphate dehydrogenase (*GAPDH*) and the ribosomal protein L32 (*RPL32*) were used as house-keeping genes for CaSki and SiHa cells, respectively. Primer sequences and probes’ numbers from the universal probe library (Roche, Germany) were as follows: *GAPDH* (probe 60): agc cac atc gct gag aca c/gcc caa tac gac caa atc c; RPL32 (probe 17): gaa gtt cct ggt cca caa cg/gag cga tct cgg cac agt a; *E6*-HPV16 (probe 115): tgt ttc agg acc cac agg a/ttg ttt gca gct ctg tgc at; *E7*-HPV16 (probe 63): caa ctg atc tct act gtt atg agc aa/cca gct gga cca tct att tca; *KCNH1* (probe 49): cct gga ggt gat cca aga tg/cca aac acg tct cct ttt cc; *TNFα* (probe 29): cag cct ctt ctc ctt cct ga/gcc aga ggg ctg att aga ga; *IL-6* (probe 40): gat gag tac aaa agt cct gat cca/ctg cag cca ctg gtt ctg t; *VIM* (probe 16): aaa gtg tgg ctg cca aga ac/agc ctc aga gag gtc agc aa; *IL-1β* (probe 78): tac ctg tcc tgc gtg ttg aa/tct ttg ggt aat ttt tgg gat ct. Real time PCR amplifications were carried out on a LightCycler^®^ 480 Instrument (Roche), according to the following protocol: activation of Taq DNA polymerase and DNA denaturation at 95 °C for 10 min, proceeded by 45 amplification cycles of 10 s at 95 °C, 30 s at 60 °C, and 1 s at 72 °C.

### 4.6. Induction of Tumors in Athymic Mice and Therapeutic Protocol

This study was approved by the Internal Committee for the care and use of laboratory animals (CICUAL, protocol number BRE-1920-18-21-1) at the Instituto Nacional de Ciencias Médicas y Nutrición Salvador Zubirán. In addition, all experimental procedures were performed according to current and valid national and international rules, including the Official Mexican Norm 062-ZOO-1999 and the Canadian Council on Animal Care guidelines. Six-week-old female athymic nude mice (BALB/c homozygous, Crl:NU(NCr)-Foxn1nu, Charles River Laboratories, Wilmington, MA, USA) were maintained under controlled temperature, humidity, and constant temperature (25 °C ± 1 °C), light/dark cycles of 12 h, with sterile food (standard irradiated 5053) and water ad libitum. The cervical cancer cells SiHa and CaSki were subcutaneously xenografted (2 × 10^6^ cells/0.1 mL of non-supplemented medium) into the lower limb of each mouse. Mice were randomly divided into two groups when the tumors reached a palpable mass of ~5 mm in diameter. They received either vehicle (0.1% of DMSO/day) or AM (8 mg/kg of body weight/day) in the drinking water for four weeks. To determine toxic effects of the drug, mice were weighed three times per week. Long and width of the tumor were measured with a caliper to calculate the tumor volume, which was accomplished using a standard formula (length × widht^2^/2). At the end of the experiments, mice were euthanized by cervical dislocation under anesthesia (sodium pentobarbital 80 mg/kg i.p.), and the tumors were divided in two parts, one of which was stored in Trizol reagent for gene expression analysis, and the other was fixed in formaldehyde for immunohistochemical staining.

### 4.7. Immunohistochemistry

The tumoral tissue that was fixed in formaldehyde (10%) and paraffin-embedded was cut into sections of two-micrometers, which were placed on glass coverslips, dewaxed, and rehydrated using standard protocols. Antigen retrieval was accomplished by autoclaving in retriever citrate solution (Bio SB (Goleta, CA, USA)). Tumor slides were blocked with immunodetector peroxidase blocker (Bio SB (Goleta, CA, USA)) and incubated for 1 h with a Ki-67 rabbit monoclonal antibody (1:100, Bio SB 5713), a cellular marker for proliferation. After washing, the slides were sequentially incubated with Immuno-Detector Biotin-Link and Immuno-Detector horseradish peroxidase (HRP) label (Bio SB (Goleta, CA, USA)) for 10 min each. Staining was completed with diaminobenzidine (DAB), and slides were counterstained with hematoxylin. Images were taken with a conventional microscope.

### 4.8. Statistical Analysis

Statistical differences were determined by one-way ANOVA followed by appropriate post hoc tests using a specialized software package (SigmaStat 3.5, Jandel Scientific, San Jose, CA, USA). Differences were considered statistically significant at a *p* value < 0.05.

## 5. Conclusions

The phytochemical AM induces cell cycle arrest, cell death, and tumor growth inhibition in cervical cancer by downregulating HPV E6/E7 and *KCNH1* oncogenes gene expression, highlighting its potential as an effective antitumoral agent for this neoplasia. These results provide the basis for further studies to test this natural compound per se as a chemopreventive cervical cancer agent or as an adjuvant for conventional cervical cancer therapy.

## Figures and Tables

**Table 1:** Cell proliferation IC_50_ values of AM in cervical cell lines bearing different HPV viral load.

Cell Line	IC_50_ (µM)
C33a	5.35 ± 0.07
HeLa	5.00 ± 0.07
SiHa	4.32 ± 0.16
CaSki	3.77 ± 0.23

Inhibitory concentrations at 50% (IC_50_) of α-mangostin (AM) to inhibit cervical cancer cell proliferation. The results are the mean ± SEM calculated from at least three independent experiments.

**Table 2:** The AM inhibitory effects on *E6/E7*-HPV16 oncogene mRNA expression.

Cell Line	*E6*-HPV16 (% of Inhibition)	*E7*-HPV16 (% of Inhibition)
SiHa	32 ± 17	26 ± 14
CaSki	66 ± 12 *	89 ± 6 *

Effect of α-mangostin (AM) 10 µM on the percentage of inhibition of *E6*- and *E7*-HPV16 gene expression, the data of the cells treated with the vehicle were considered as 0% of inhibition. The results are the mean ± SEM of the percentage of inhibition of each oncogene. At least three independent experiments were carried out. The values highlighted in bold were statistically significant. * *p* < 0.05 vs. vehicle.

**Table 3:** The AM effect upon *KCNH1* gene expression in human cervical cancer cell lines.

AM (µM)	*KCNH1* mRNA Inhibition (%)	
	SiHa	CaSki
0	0 ± 12	0 ± 8
4	16 ± 26	13 ± 18
6	16 ± 19	30 ± 19 *
8	17 ± 30	33 ± 22 *
10	18 ± 19	53 ± 19 *

The effect of α-mangostin (AM) on *KCNH1* gene expression. The results are presented as the mean ± SEM of percentage of inhibition of vehicle-treated cells (0 µM) and AM treated cells. *n* ≥ 3 independent experiments. The values highlighted in bold were statistically significant. * *p* < 0.05 vs. vehicle.

**Table 4:** Expression of *KCNH1* and HPV-oncogenes in cervical cancer cells.

Cell Line	*KCNH1*	HPV	References
C33a	+	-	[50]
HeLa	+	Sequences of HPV-18	[7]
SiHa	+	1–2 copies of HPV-16	[51]
CaSki	+	~600 copies of HPV-16, sequences of HPV-18	[50]

Expression (+) of *KCNH1*. Different profile of the human papilloma virus (HPV) expression, obtained from the website at atcc.org (accessed on 9 December 2022). C33a cells do not express HPV.

## Data Availability

Not applicable.
